# Characterization of Nanospheres Containing *Zanthoxylum riedelianum* Fruit Essential Oil and Their Insecticidal and Deterrent Activities against *Bemisia tabaci* (Hemiptera: Aleyrodidae)

**DOI:** 10.3390/molecules23082052

**Published:** 2018-08-16

**Authors:** Karla de Castro Pereira, Eliane Dias Quintela, Daniel José da Silva, Vinicius Alves do Nascimento, Dannilo V. M. da Rocha, José Francisco Arruda e Silva, Moacir Rossi Forim, Fabiano Guimarães Silva, Cristiane de Melo Cazal

**Affiliations:** 1 Goiano Federal Institute of Education, Science and Technology-Campus Rio Verde, Rod. Sul Goiana, Km 01, 75901-970 Rio Verde, Brazil; karla.castro@ifgoiano.edu.br (K.d.C.P.); fabiano.silva@ifgoiano.edu.br (F.G.S.); 2 Federal University of Goias, Avenida Esperança, s/n, Campus Samambaia, 74690-900 Goiânia, Brazil; 3 Brazilian Corporation of Agricultural Research-Embrapa Rice and Bean, Rodovia GO-462, Km 12, Fazenda Capivara, Zona Rural, Caixa Postal 179, 75375-000 Santo Antônio de Goiás, Brazil; eliane.quintela@hotmail.com (E.D.Q.); dannilovono@hotmail.com (D.V.M.d.R.); j.chikosilva@hotmail.com (J.F.A.e.S.); 4 Federal Institute of Education, Science and Technology of Southeastern Minas Gerais-Campus Barbacena, Rua Monsenhor José Augusto, n 204, Bairro São José, 36205-018 Barbacena, Brazil; danieljose95@ymail.com (D.J.d.S.); viniciusnasciment@yahoo.com.br (V.A.d.N.); 5 Department of Chemistry, Federal University of São Carlos, Rod. Washington Luiz, km 235, CP 676, 13565-905 São Carlos, Brazil; mrforim@gmail.com

**Keywords:** GC-MS, nanoprecipitation method, whitefly, PCL

## Abstract

The aim of our study was to produce and characterize poly-ε-caprolactone (PCL) nanospheres containing essential oils from *Zanthoxylum riedelianum* fruit and to evaluate their stability gains as well as their insecticidal and deterrent activities against whitefly (*Bemisia tabaci*). The PCL nanospheres exhibited a homogeneous spherical morphology, with particle diameters between 106.7 nm and 129.2 nm, pH of approximately 6, zeta potential (ZP) lower than −19.0 mV and encapsulation efficiency higher than 98%. Only 43% of the nanoencapsulated essential oil (NSEO) was degraded in response to ultraviolet light, whereas the essential oil (EO) degraded by 76% over the same period. In a free-choice test, the NSEO and EO reduced the number of whitefly eggs by approximately 70%. NSEO and EO at 1.5% killed 82.87% and 91.23% of 2nd-instar nymphs of whitefly, respectively. Although NSEO displayed lower insecticidal activity, it offers a greater advantage over the free EO, due to protection conferred by polymer against photodegradation. Therefore, its usage may optimize the maintenance of essential oils in the field through photoprotection and controlled release. Our results suggest that the EO of *Z. riedelianum* fruit can be used for *B. tabaci* management strategy; nevertheless, the benefits of NSEO require further evaluation at the field level.

## 1. Introduction

Every year, an estimated two million tons of chemical pesticides are used on crops worldwide [1]. Their excessive use has caused significant environmental damage and compromised public health due to their toxic, non-biodegradable properties and the accumulation of residues in soil, water, and food [2,3]. Thus, studies focusing on alternatives to pesticides have increased in recent years [4,5,6]. Plant essential oils are promising alternatives due to their biodegradability, lower toxicity to nontarget organisms, and maintenance of ecological balance [7]. For this reason, essential oils have been termed “green pesticides” [3].

Essential oils are isolated from plants as a complex of volatile, lipophilic, strongly odiferous compounds resulting from the secondary metabolism. In plant secretions, several of its constituents are commonly used to attract pollinators, protect against heat or cold, and defend against predators and/or parasites [3]. Their components can disrupt cuticular waxes and membranes in the integument of insects, leading to desiccation, and also act on the digestive and neurological enzymes (e.g., inhibiting acetylcholinesterase) of insects [8]. These mechanisms of action favor their use in the control of commercially important pests such as *Bemisia tabaci*, popularly known as the whitefly. 

*Bemisia tabaci* is responsible for large losses each year in crops such as bean, soybean, tomato, cabbage, and ornamental plants [9,10]. Direct damage is caused by feeding on the plant’s phloem sap and injecting toxins, debilitating the plants. Sap consumption causes indirect damage, as the sugary residue left on the plant may foster the growth of saprophytic fungi, whose soot-like aspect may decrease the plant photosynthetic area and the commercial value of the crop [11]. Moreover, *B. tabaci* is a vector of more than 300 species of virus [12,13], including Begomovirus (family Geminiviridae), Crinivirus (family Closteroviridae), and Carlavirus (family Betaflexiviridae) in Brazil. Begomoviruses have significantly hindered tomato and bean cultivation, particularly in warm, dry climates [14,15]. 

Several studies have investigated the efficacy of essential oils in the control of whitefly (*B. tabaci*). *Thymus vulgaris*, *Pogostemon cablin*, and *Corymbia citriodora* essential oils effectively reduced oviposition, egg hatching, and nymphal survival [16]. Essential oils from *Micromeria fruticosa*, *Nepeta racemosa*, and *Origanum vulgare* caused adult mortality [17]. Emilie et al. [18] evaluated the repellent, irritant, and toxic effects of 20 essential oils, seven of which were toxic and irritant to *B. tabaci*. Essential oils from *Acorus tatarinowii* Schott, *Heracleum hemsleyanum* Diels, and *Stemona japonica* (Blume) Miq. exhibited fumigant and contact toxicity, in addition to deterring *B. tabaci* oviposition [19]. The genus *Zanthoxylum* (family Rutaceae) encompasses a group of more than 200 species with pantropical distribution [20] and is known for its pharmacological and pesticidal properties. The anti-inflammatory and analgesic activities of *Zanthoxylum riedelianum* have been reported in the literature [21]. Costa et al. [22] demonstrated the repellent effect of the essential oil of *Z. riedelianum* fruit against *B. tabaci*. 

Despite their potential, the use of essential oils in agriculture is limited due to their high volatility, photoinstability, and thermolability, which contribute to the need for high doses and/or numerous applications. Therefore, an association between essential oils and nanotechnology may be advantageous by decreasing the photo- and thermo-degradation of the essential oil active compounds [23]. Nanoparticles (NP), which generally range from 10 nm to 1000 nm in size, contain dissolved or encapsulated active compounds within their polymer matrix. Because NPs are solid, compound mobility is lower than that in liquid medium, generating a controlled release system that depends on compound diffusion through the matrix and/or on polymer erosion [24]. NPs have been shown to be efficient in the release of active molecules from the active site in the polymer matrix. Thus, the slow release and the enhanced oil dispersion in aqueous media conferred by NPs may physically stabilize essential oil active molecules and increase their persistence in the field, favoring a lower number of product applications [23,25].

To avoid environmental impacts, nanoparticle polymer coatings need to be biodegradable and innocuous. Poly (ε-caprolactone) (PCL) is an aliphatic polyester with such characteristics; furthermore, it is biocompatible. PCL has been used in the controlled release of active pharmaceutical compounds since the 1970s [26,27]. These factors led to an interest in combining PCL with biopesticides for agricultural applications.

Considering the advantages of nanotechnology for essential oil-based biopesticide production, the aim of the present study was to develop and characterize PCL nanospheres containing the essential oils of *Z. riedelianum* fruit. Furthermore, nanoencapsulated essential oils were evaluated in terms of their stability gains and their insecticidal and deterrent activities toward *B. tabaci* (Gennadius, 1889) (Hemiptera: Aleyrodidae).

## 2. Results and Discussion

### 2.1. Chemical Composition of the Z. riedelianum Fruit Essential Oil

The essential oils of *Z. riedelianum* fruit obtained via hydrodistillation exhibited a light yellowish color, characteristic odor, density lower than that of water, and an average yield of 1.77 ± 0.06% (*w*/*w*).

Twenty-three compounds were detected, of which 22 were identified. Major compounds included limonene (29.22%), β-myrcene (22.79%), bicyclogermacrene (18.13%), and germacrene D (14.40%) (Table 1). Costa et al. [22] identified γ-elemene (21.2%), germacrene D (14.2%), sabinene (11.9%), and limonene (11.3%) as the main components of the *Z. riedelianum* fruit essential oil. Other compounds similar to those described herein were described by Costa et al. [22], including bicyclogermacrene, spathulenol, β-caryophyllene, germacrene B, and α-pinene, among others—albeit in different amounts. Although some compounds corroborate the existing literature, the existing differences are determined by several factors, such as the plant development stage, genetic traits, soil composition, climate, and nutrition [28,29,30]. Such factors induce significant changes in secondary metabolite production, influencing the content and chemical composition of the essential oil. 

### 2.2. Validation of the Z. riedelianum Fruit Essential Oil Analytical Quantification Method

Correct determination of the encapsulation efficiency (EE%) and UV light-induced degradation requires validation of the analytical method.

Analytical curves were obtained by linear regression, and the line equation (y = 4.8646x + 0.0609, where y is the mean absorbance value, and x is the concentration of the essential oil in mg/mL) was linear (*n* = 3) in the working range, with a correlation coefficient (r^2^) of 0.999.

Repeated intraday (*n* = 3) and interday (*n* = 9) precision values were ≤0.4, demonstrating that the analytical method was highly precise. The mean accuracy value was 100.4 ± 0.7; thus, a high degree of agreement was found between the experimental and theoretical values (Table 2).

The detection (DL) and quantification (QL) limits were 0.0059 mg/mL and 0.018 mg/mL, respectively. The QL was lower than the first point of the calibration curve (≤0.05 mg/mL). The method developed for *Z. riedelianum* essential oil quantification behaved linearly and showed accuracy and precision across the entire working range.

### 2.3. Physicochemical Characterization of Nanospheres

The nanoprecipitation method used to encapsulate the *Z. riedelianum* fruit essential oils was based on precipitation of the polymer after addition of a nonsolvent to a solution containing the polymer. Four mechanisms occurred, namely, supersaturation, nucleation, growth by condensation, and growth by coagulation, leading to the formation of nanoparticles [31]. Those authors also noted that this method shows good reproducibility in the laboratory and constitutes a good strategy for nanoparticle production at an industrial scale. Other advantages offered by the method include the simple execution and the use of solvents relatively less toxic than many others, such as ethanol and acetone [32].

The nanoprecipitation method was efficient in encapsulating *Z. riedelianum* fruit essential oils since the colloidal suspensions exhibited EE% ≥ 98.66% (Table 3). Previous studies reported similar results using the same method and the PCL polymer; for instance, EE% was ~99% for rosemary essential oil [33] and greater than 96% for *Z. rhoifolium* leaf essential oil [34]. Forim et al. [23] used a new, nanoprecipitation-based method capable of encapsulating 100% of neem extract.

The stability of the nanoparticulate systems was maintained by employing a high hydrophilic–lipophilic balance (HLB) surfactant—Tween^®^80—which has the purpose of preventing particle coalescence and diffusion of the encapsulated active substance. The low HLB surfactant, Span^®^60, present in the organic phase, is necessary to obtain a population of nanospheres with a small and homogeneous size [35,36]. The stability and uniformity of the suspensions were evaluated by their pH, zeta potential (ZP), particle diameter (PD), and polydispersity index (PdI) values.

The pH values of the formulations decreased with increasing amounts of essential oil (Table 3) but remained between 6.19 and 6.66, a range considered satisfactory [37]. Previous studies on the encapsulation of plant extracts and essential oils using PCL as a polymer revealed pH values between 4 and 6, corroborating our results [23,34]. Low pH values (<4.0) indicate degradation of the polymer and/or of some sample component. The polymer itself (PCL) can decrease pH values due to the relaxation of its polymer chains, resulting in the exposure of carboxylic acid groups. However, acidic pH values can lead to polymer hydrolysis, yielding unstable suspensions and generating sediments in sample vials [37]. No sediment formation was observed before any analysis or bioassay was conducted, indicating the stability of the nanoformulations, which was also confirmed by the zeta potential (ZP) values.

The ZP (ζ) measures the electrostatic repulsion of the particles’ electric charges. ZP values ≥30 mV or ≤−30 mV indicate good physicochemical stability of the suspension, since the repulsion between charged particles is higher, preventing coalescence driven by occasional collisions between adjacent nanoparticles [38,39,40]. In the present study, no significant difference existed between the ZP values of the samples (Table 3), with values around −24 mV; the sole exception was NS2, whose ZP was −19.0 ± 2.4 mV. Similar results were obtained in the encapsulation of rosemary essential oil, with ZP ≤ −19.9 [33]. PCL nanospheres containing *Z. rhoifolium* leaf essential oil presented ZP ≤ −26.12 [34].

According to Woodruff and Hutmacher [26], nanosphere size can vary between 10 and 1000 nm. All suspensions in the present study presented nanospheres within this diameter range, with values between 106.7 and 129.2 nm (Table 3). A significant difference existed in particle diameter values; however, the increase in the amount of essential oil (mg) did not influence this feature. According to Schaffazick et al. [40], average nanoparticle diameters fall between 100 and 300 nm regardless of the preparation method (e.g., solvent displacement (nanoprecipitation), emulsion polymerization, or emulsification-diffusion), thus corroborating our results. Similar results were also obtained by Pinto et al. [41], who reported a mean diameter of 173.6 nm for PCL nanocapsules containing *Lippia sidoides* leaf essential oil formed by solvent emulsification-diffusion. Nanocapsules containing rosemary essential oil formed by nanoprecipitation showed PDs of approximately 220 nm [33]. Yang et al. [42] obtained nanoparticles containing garlic essential oil with approximately 233 nm in diameter using the dispersion-fusion technique.

Another important parameter in the evaluation of suspension quality is the polydispersity index (PdI), which provides information on the size homogeneity of suspended nanoparticles. Some researchers classify PdI values up to 0.3 as monodispersed [33,43,44]. No significant difference existed in the PdI values, with values close to 0.2 (Table 3). These results indicate a narrow range of nanosphere sizes. 

### 2.4. Morphology of the Z. riedelianum Fruit Essential Oil-Containing Nanospheres

**Figure 1:**
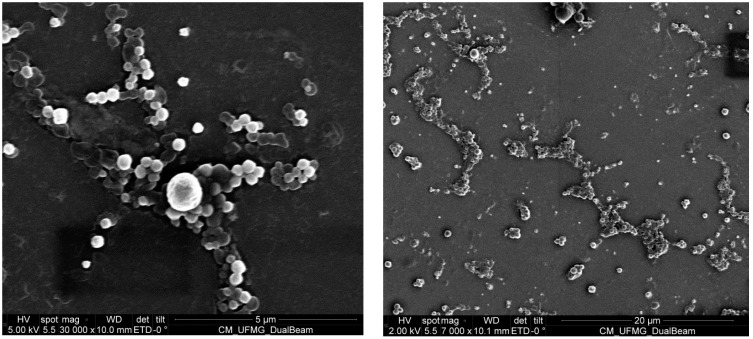
SEM of PCL nanosphere suspensions containing *Z. riedelianum* fruit essential oil.

Scanning electron microscopy (SEM) revealed spherical nanoparticles with regular shape and surfaces (Figure 1). The images also showed a system with nanospheres of relatively homogeneous sizes, corroborating the PdI values. Similar results regarding nanoparticle morphology and homogeneity of essential oil-containing suspensions have been described in the literature [23,33,42,45].

### 2.5. UV Light-Accelerated Degradation

**Figure 2:**
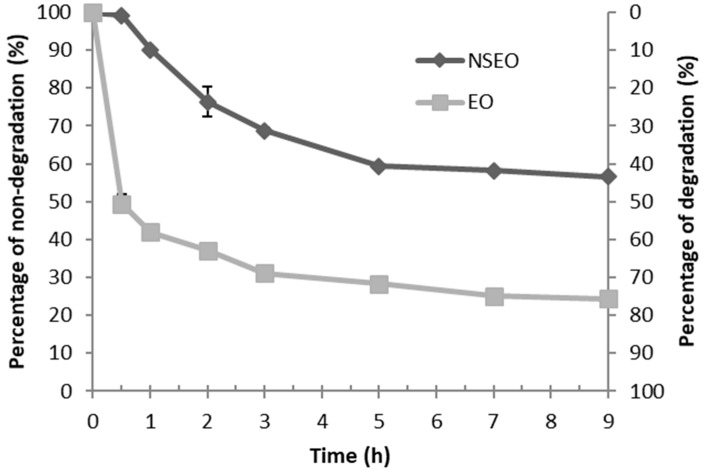
Degradation profile of the *Z. riedelianum* fruit essential oil (EO) and nanoencapsulated essential oil (NSEO) after UV light radiation.

After 9 h of UV radiation exposure, EO and NSEO *Z. riedelianum* fruit reached degradation levels of 76% and 43%, respectively (Figure 2). A previous study reported *Z. rhoifolium* leaf essential oil photodegradation levels of 94.33% and 44.76% after 7 h of exposure to UV radiation for EO and NSEO, respectively [34]. These results demonstrate the important roles played by nanospheres in protecting the essential oil against photodegradation. 

Product persistence in the field is a known agricultural challenge. Products are easily degraded by light or microorganisms, or they undergo hydrolysis. Photodegradation is the most common process in pesticides [46]. In a study involving chitosan-coated beeswax-based nanoparticles containing deltamethrin, 37.3% of the product had not been degraded after 24 h of UV light irradiation, whereas 85% of the free form degraded under similar conditions [47]. 

Natural products are even more photounstable and thermolabile than synthetic insecticides [48], limiting the use of essential oils in agricultural applications, for instance. According to the results described herein, nanotechnology reduced photodegradation, offering an advantage when compared to the free form.

### 2.6. Deterrence of EO and NSEO Z. riedelianum against B. tabaci

In all bioassays with nymphs and adults, it was not observed phytotoxicity by the essential oil on bean leaves, even at higher concentrations of 1% and 1.5%.

The deterrent effect of the essential oil against adult whiteflies was evaluated through oviposition on bean leaves in free-choice and no-choice tests. 

In the free-choice test, all tested concentrations of EO and NSEO significantly reduced the number of eggs relative to the controls with Tween and empty nanospheres (formulation without the essential oil), respectively (Table 4). The reduction in the number of eggs with both forms of the essential oil was similar to that of the insecticide treatment (Table 4). The number of eggs did not differ between NSEO and EO treatments over the tested concentration range. The data suggest that the nanoencapsulation process did not affect the whitefly oviposition-deterrent effect of *Z. riedelianum*. According to the oviposition index, the NSEO and EO and the insecticide spiromesifen were whitefly oviposition deterrents at all tested doses (Table 4).

In the no-choice test, the number of eggs was significantly lower in the treatment with EO at all doses than the control (Tween) (Table 4). Moreover, all tested doses of the EO were oviposition-deterring according to the oviposition index (Table 4). NSEO reduced the number of eggs only in treatments at 0.25% and 1.5% compared to the NS control (empty nanosphere—formulation without essential oil). The oviposition-deterrent effect of NSEO was observed only at the 0.25% concentration (Table 4). Similar to the free-choice test, the no-choice test showed no differences in the number of eggs between NSEO and EO at all concentrations tested. Similar results were observed by Costa et al. [22]. The *Z. riedelianum* fruit essential oil decreased the number of *B. tabaci* eggs on tomato leaves by 85.7% and 94.2% for the 1.0% and 1.5% concentrations, respectively. Christofoli et al. [34] also demonstrated that *Z. rhoifolium* Lam. leaf EO at 0.5% or 1.0% reduced *B. tabaci* oviposition by 71% and 77%, respectively, three days after treatment application. These authors also showed that the *Z. rhoifolium* nanoencapsulated essential oil at the 2% and 5% doses reduced the number of eggs by 95.6% and 93.9%, respectively, when compared to the control (water). The greater oviposition reduction obtained with *Zanthoxylum* essential oil by Christofoli et al. [34] compared to the present study may stem from the variation in the chemical composition of the essential oil. The quality and quantity of essential oil components may vary according to plant genetics, climate, soil composition, plant organ, age, and stage of the vegetative cycle, etc. [30,49,50,51].

The reduction in whitefly oviposition on bean leaves treated with *Z. riedelianum* NSEO and EO may have been caused by different factors. These factors include the lipidic nature of the essential oil, which may have affected a glue-like substance secreted by whiteflies used to attach the egg pedicel to the host plant tissue [52,53] and prevented the eggs from attaching to the plant, causing them to fall. Yang et al. [16] also showed a decrease in the number of eggs deposited by female whiteflies on tomato leaves treated with *Thymus vulgaris* L., *Pogostemon cablin* (Blanco) Benth., and *Corymbia citriodora* essential oils.

Saad et al. [54] attributed the decrease in *B. tabaci* oviposition to the volatile compounds found in citronella (*Cymbopogon nardus* L.) essential oil, which may have affected the development of the female whitefly, reducing its reproductive capacity. An example of this activity was reported by Rao et al. [55], who found that *Dysdercus koenigii* nymphs treated with *Artemisia annua* essential oil showed ovarian development issues, decreasing the number of produced oocytes with a consequent influence on egg production.

Frequent changes in feeding sites due to the persistence of the essential oil in the plant tissue are another factor that may have influenced whitefly oviposition. This may reduce phloem sap suction, leading to a decrease in the number of mature eggs for deposition [16,56]. Reduced feeding and oviposition can also be influenced by the detection of some volatile substances by chemoreceptors present in the insect’s tarsi, deterring oviposition [52,57]. 

### 2.7. Mortality of Second-Instar B. tabaci Nymphs

**Figure 3:**
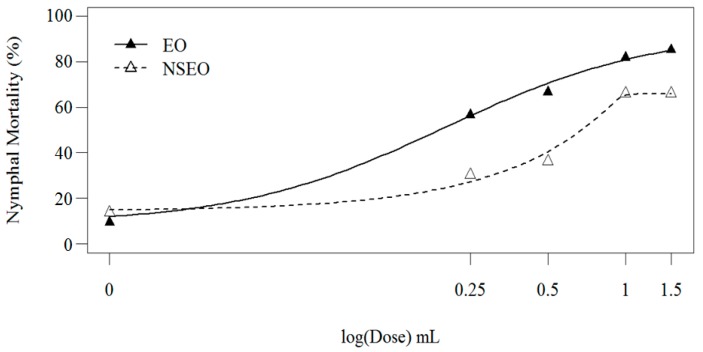
Second-instar nymphal mortality 11 days after treatment of bean leaves with *Z. riedelianum* fruit essential oil (EO) and nanoencapsulated essential oil (NSEO) (Bioassay 1).

In the first experiment, EO and NSEO *Z. riedelianum* had a significant effect on the mortality of nymphs (56.53–85.20% and 30.23–66.18%, respectively) (Figure 3). Nymphal mortality gradually increased with essential oil concentrations, regardless of form. A log-logistic model provided the best fit for both EO and NSEO (*R*^2^ = 89.80%) (Figure 3). When the two curves were compared using Wilcoxon’s test, the essential oils were significantly different (*p* < 0.001).

A lower activity of NSEO was also observed by Carvalho et al. [59]. PCL nanoformulations containing neem oil killed 18.9% of 1st-instar *B. tabaci* nymphs, compared to 60% caused by the free form. In turn, Carvalho et al. [60] obtained different results for two types of nanocapsules containing Azamax^®^ 1.2 EC, a commercial formulation enriched with azadirachtin and 3-tigloylazadirachtol. For the same concentration of Azamax, PCL, and poly-β-hydroxybutyrate (PHB) nanocapsules caused the mortality of 98% and 70.6% of *B. tabaci* nymphs, respectively. This variation in results was attributed to environmental conditions, such as greater photoperiod, light intensity, or thermal amplitudes. These factors may have accelerated polymer breakdown, with a concomitant faster release of the active compound. 

The lower activity of the NSEO compared to EO forms may also be related to the type of formulation used for nanoparticle preparation and/or to the environmental conditions. According to Soppimath et al. [24], the release of active compounds by nanoparticles, as well as nanoparticle biodegradation, is important for a successful formulation. Release rates depend on the desorption of the active principle from the surface of the nanoparticles, their diffusion through the polymer matrix, erosion of the matrix, and erosion-associated diffusion. Therefore, diffusion and biodegradation are essential processes for the controlled release system. Depending on the formulation, the active compounds may remain entrapped in the polymer matrix, reducing product activity. De Oliveira et al. [61] prepared *Lippia sidoides* essential oil-based nanoparticles with different ratios of alginate (ALG) and cashew gum (CG): 1:3, 1:1, and 3:1. The nanoparticles formed with a 3:1 ALG:CG ratio exhibited a lower, more controlled release of the essential oil (45% in 50 h), whereas an ALG:CG 1:3 ratio released 65% of the essential oils after 5 h. Thus, the nanoparticle release profile depended on the ALG:CG ratio.

**Figure 4:**
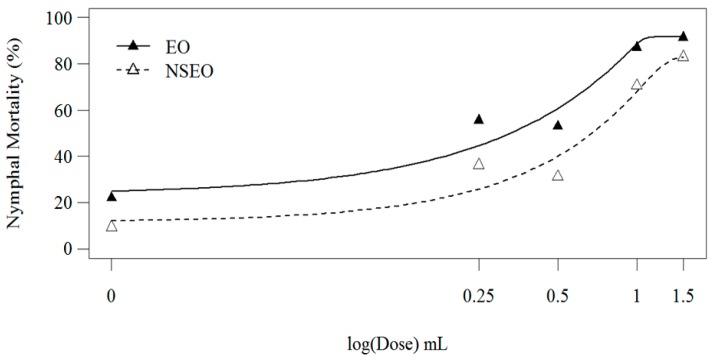
Second-instar nymphal mortality 11 days after treatment of bean leaves with *Z. riedelianum* fruit essential oil (EO) and nanoencapsulated essential oil (NSEO) (Bioassay 2).

The second experiment was conducted with the same nanoformulations but at a lower relative humidity (48.41 ± 7.78%). Nymphal mortality also increased with increasing concentrations of essential oil (Figure 4). 

According to Wilcoxon’s non-parametric test, the two curves were significantly different (*p* < 0.001), where the EO killed more 2nd-instar nymphs than the NSEO. Although this finding corroborates the first experiment, the efficiency of the NSEO at the 1.5% dose increased in the second test (*p* < 0.05). The EO form of *Z. riedelianum* fruit essential oil at concentrations of 0.25, 0.5, 1.0, and 1.5% caused mortality of 55.57, 64.30, 86.98, and 91.23% of the nymphs, respectively. The NSEO at concentrations of 1.0% and 1.5% killed 70.55% and 82.87% of the nymphs, respectively. The lowest concentrations, 0.25% and 0.5%, did not differ significantly, reducing nymph numbers by just 30.93% and 31.20%, respectively. The highest dose (1.5%) of both NSEO and EO were statistically similar to the chemical insecticide cyantraniliprole (Table 5).

The higher efficiency of the essential oil in the second experiment is likely related to the environmental conditions, such as the lower relative humidity, corroborating Carvalho et al. [60]. Many essential oil components are photounstable and thermolabile [48]. These characteristics limit the use of essential oils in agriculture due to a high exposure to UV radiation and temperature. However, nanoencapsulation improves the efficiency and persistence of these natural products [62]. Therefore, although the percent nymphal mortality was higher in free essential oil form, nanoencapsulates may become more advantageous by reducing photo- and thermo-degradation issues of the active compound, possibly maintaining their activity for longer periods of time [23]. 

Nanoencapsulation protects the active compounds in the essential oil from environmental factors (e.g., light and heat) that affect their molecular structures. UV radiation with wavelength (λ) near 350 nm is capable of breaking bonds between carbon atoms in organic molecules, thereby generating radicals that accelerate the oxidation rate of the compounds [63]. This effect can be minimized or retarded by coating polymers such as PCL, as these will act as a protective barrier, leading to a slow release of the compounds adsorbed into their polymer matrix as they degrade.

Other studies have also demonstrated the efficiency of nanoparticles containing plant extracts in pest control. Forim et al. [23] used a new technique to prepare nanocapsules containing *Azadirachta indica* (neem), based on the solvent displacement method. These nanocapsules caused the mortality of 100% of *Plutella xylostella* larvae. The nanoparticles containing *Allium sativum* L. essential oil controlled *Tribolium castaneum* (red flour beetles) with >80% efficacy, whereas the free form reduced the beetles by only 11% after five months of product application [42]. Nanoemulsions of three species of the genus *Achillea* also presented insecticidal activity against *T. castaneum* [64].

Some hypotheses can be formulated to explain nymphal susceptibility to *Z. riedelianum* essential oil: (1) Whiteflies, for instance, possess a waxy layer on their body, which hinders insecticide penetration [65]. Essential oils thus have the advantage of disrupting this layer and acting on insect metabolism in several ways, including inducing mitochondrial membrane depolarization [8]. The decrease in membrane potential (depolarization) affects the calcium ion cycle and reduces the pH gradient, affecting the proton pump and ATP pool [7,66,67]. These changes render the mitochondrial membrane abnormally permeable, which results in oxidative stress and bioenergetic failure, leading to necrosis- and apoptosis-induced cell death [68]. (2) In insects, acetylcholinesterase (AChE) inhibition and acetylcholine accumulation leads to excitation and death [69]. Some essential oil active compounds, such as monoterpenes, are competitive inhibitors of acetylcholinesterase (AchE). The inhibition mechanism may be related to the chemical structure of the monoterpene, comprising one methyl allyl group and a double bond [70]. Limonene, one of the major components of the *Z. riedelianum* fruit essential oil, inhibited 87.42% of the AchE activity [71]. The compounds α-pinene and β-caryophyllene—alone or in combination—also inhibited AchE [72]. Moreover, monoterpenes can act lethally by interfering with the neuromodulator octopamine or the GABA-bound chloride channels [73]. This feature grants essential oils a selective toxicity against insects since mammals (vertebrates) do not have octopamine receptors. (3) The insecticidal activity of the *Z. riedelianum* fruit essential oil can also be attributed to the presence of compounds such as limonene and β-myrcene. Hollingsworth [74] showed that treatments containing 1% limonene caused 99% mortality of *Aleurodicus dispersus* whiteflies. Five mg/cm^2^ of β-myrcene induced 100% mortality of *Blatella germanica* adult females [75]. Prieto et al. [76] attributed the insecticidal activity of *Z. rhoifolium* against *Sitophilus oryzae* to β-myrcene and β-phellandrene. 

In our study, twenty-two compounds were identified in *Z. riedelianum* fruit essential oil (Table 1). Probably, the biological effects of this essential oil was from the synergism between all molecules, from the major molecules, or be modulated by the minor molecules as observed in others studies with essential oils [7,77]. Zarrad et al. [71] reported that *Citrus aurantium* L. essential oil was more toxic to *B. tabaci* adults than pure limonene. This result revealed the additive or synergistic effect of the components of *C. aurantium* oil. The combination of different substances has the advantage of reducing the resistance of insect pests, as they decrease selection pressure, which would not occur if a single compound was used. These factors reinforce the advantages of using essential oils compared to the isolated component.

In summary, the *Z. riedelianum* fruit essential oil was effective as an insecticide and oviposition deterrent against *B. tabaci*. Although some results have showed that the EO killed more second-instar nymphs than the NSEO form, it presented similar activity to the synthetic insecticide cyantraniliprole. The nanoencapsulated form demonstrated similar efficiency to the EO form in the free-choice and no-choice tests for whitefly oviposition deterrence. Nanoencapsulated essential oils offer a greater advantage over the free essential oil forms, with protection conferred by the PCL polymer against photodegradation (results described herein). Through the nanoencapsulation, it is possible to optimize the maintenance of the essential oil in the field reducing the requirement of extra applications that clearly impact final cost of the crops. Given that the nanoformulated products usually display differential bioavailability in the field, a greater stability implies a greater permanence in the cultivation [47,78]. Moreover, once essential oils are volatiles, they are easily spread in the environment, losing their ability to control insects, so we need a way for them to be fixed in the field. Thus, nanoformulation may be an option to solve this sort of practical issue. Therefore, the results presented here may be further used as the foundation to the development of commercial products using essential oils such as from fruits of *Z. riedelianum*.

## 3. Materials and Methods

### 3.1. Obtaining the Essential Oil

*Zanthoxylum riedelianum* fruits were collected in the rural area of two municipality namely Orizona (17°00′16″ S; 48°04′15″ W) and Iporá (16°26′44.29″ S, 51°8′0.61″ W, both in Goiás (GO) state, Brazil. The exsiccates were deposited in the herbarium of the Federal University of Goiás [Universidade Federal de Goiás] in Goiânia, GO, Brazil (16°36′23.7″ S; 49°15′50.5″ W) under n. 60213 and in the herbarium of the Goiano Federal Institute of Education, Science and Technology [Instituto Federal de Educação, Ciência e Tecnologia Goiano] in Rio Verde, GO, Brazil (17°48′19.5″ S; 50°54′21.3″ W) under n. 625, respectively. We only used fruits that had the first signs of carotenoid accumulation on the external surface—defined as the breaker stage prior to the red ripening of the fruits.

The essential oil of *Z. riedelianum* fruit was obtained by hydrodistillation for a period of 3 h using a Clevenger-type apparatus coupled with a 3-L round bottom flask. The hydrolate was centrifuged for 15 min at 2000 rpm for phase separation. The organic (less dense) phase was removed using a glass Pasteur pipette. Anhydrous sodium sulfate (Na_2_SO_4_) was added to the solution to remove excess water and was subsequently filtered. The obtained essential oil was transferred to an amber bottle and refrigerated. The yield (%) was determined by the ratio between the weight (g) of the oil obtained and the weight of fruit (g) used in the extraction. 

We performed at least three extractions of essential oil from samples harvested in the distinct points of collect. The essential oils were put together to obtain enough material for the biological assays.

### 3.2. Chemical Analysis of Essential Oil

The chemical composition of the essential oil obtained from *Z. riedelianum* fruit was analyzed via a gas chromatographer coupled to a sequential mass spectrometer (MSTQ8030 Shimadzu, Shimadzu, Tokyo, Japan) (GC-MS/MS), equipped with an auto-injector (Combi PAL AOC-5000, CTC Analytics, Aargau, Switzerland), Restek Rtx-5ms (Restek, Bellefonte, PA, USA) silica-fused column (30 m × 0.250 mm × 0.25 μm), and electronic impact ionization detector (70 eV) ( Shimadzu, Tokyo, Japan). The separation data and their treatment were performed using the software CGMS Real-Time Analysis^®^. The initial temperature was set to 60 °C, followed by ramping along two temperature gradients, 3 °C/min until 200 °C and 15 °C/min until 280 °C, where the temperature remained for 1 min. The parameters used were 230 °C injector temperature, 300 °C detector temperature, 57.4 kPa injection pressure, 1:50 splitless ratio, and 43–550 *m*/*z* mass spectrometer detection range. Helium was used as carrier gas at a constant flow rate of 3 mL/min. Quantitative analysis was performed using a gas chromatographer equipped with a flame ionization detector (GC-FID) (GC-2010 Shimadzu, Shimadzu, Tokyo, Japan), under the same conditions as for GC-MS. The essential oil components were identified by comparison against the NIST database and the literature based on the retention indices (RI), which were determined relative to a series of *n*-alkanes and the fragmentation pattern observed in the mass spectra.

### 3.3. Quantification of Essential Oil

UV-Vis spectrophotometry (DR/5000 UV-Vis HACH, Hach Company, Loveland, CO, USA) at 253.5 nm was used to quantify the essential oil of the *Z. riedelianum* fruits [34,79,80]. The calibration curve were obtained based on the concentrations 0.05, 0.10, 0.15, 0.20, 0.25, and 0.30 mg/mL of essential oil in hexane. 

Precision and accuracy were analyzed at concentrations of 0.06, 0.15, and 0.27 mg/mL and were expressed by the relative standard deviation (RSD) and by the relation between the mean experimental and theoretical concentrations, respectively.

The detection (DL) and quantification (QL) limits were calculated according to the standard deviation of the intercept (0.009) and the slope (4.794) of the calibration curve (0.05, 0.10, 0.15, 0.20, 0.25, and 0.30 mg/mL).

(1) $$\mathrm{DL}=\frac{3*s}{S}$$

(2) $$\mathrm{QL}=\frac{10*s}{S}$$

where *s* is the standard deviation of response and *S* is the calibration graph coefficient.

### 3.4. Preparation of Nanospheres Containing Z. riedelianum Fruit Essential Oil

The compounds used for nanosphere production were polycaprolactone (PCL) (Sigma-Aldrich, St. Louis, MO, USA), polysorbate 80 (Tween 80) (NEON), acetone PA (NEON), and span^®^60 (Sigma-Aldrich). Using the preformed polymer nanoprecipitation method (solvent displacement) proposed by Fessi et al. [81], four nanosphere colloidal suspension formulations (Table 6) were prepared in triplicate. The organic phase was prepared under mild temperature conditions (maximum 40 °C) and under stirring, consisting of PCL biopolymer, Span^®^ 60 (low-hydrophilic-lipophilic balance [HLB] surfactant), acetone PA (organic solvent), and different amounts of essential oil (mg). The organic phase was slowly poured over an aqueous phase containing distilled water and Tween^®^ 80 (high HLB surfactant) using a peristaltic pump (Watson-Marlow), moderate stirring. The mixture was left to stir for an additional 10 min for stabilization. Subsequently, the organic solvent and part of the water were eliminated via a rotary evaporator, thereby adjusting the final volume of the colloidal suspension.

### 3.5. Physicochemical Characterization of Nanospheres

The following parameters were used in the physicochemical characterization of the nanospheres: encapsulation efficiency (EE%), pH, particle diameter (PD), polydispersity index (PdI), and zeta potential (ZP).

The encapsulation efficiency was determined using the filtration-centrifugation technique. The colloidal nanosphere suspensions (1.0 mL) were transferred to tubes containing 0.22-μm pore size cellulose acetate filters (Spin-X, Corning^®^ Incorporated, Corning, NY, USA). The suspensions were subsequently centrifuged (Thoth 9300R, Thoth Equipment, Piracicaba, SP, Brazil) for 40 min at 8000 rpm at 20 °C. After centrifugation, 250 μL of the ultrafiltrate was added to 2.5 mL of hexane for liquid-liquid extraction using a vortex apparatus. The hexane fraction was analyzed by UV/Vis spectroscopy (DR/5000 UV-Vis HACH, Hach Company, Loveland, CO, USA).

The percent encapsulation efficiency (EE%) was determined by the difference between the total amount of essential oil used in the preparation of the sample and the total amount of essential oil in the ultrafiltrate via the following equation:

(3) $$EE\%=\frac{\left(B-A\right)}{B}*100$$

where *A* is the total concentration of essential oil in the ultrafiltrate (mg/mL), and *B* is the total concentration in the suspension (mg/mL).

The pH values were determined immediately after preparing the colloidal suspensions, using a potentiometer (HANNA model FT-P21, Tecnal Scientific Equipment, Piracicaba, SP, Brazil) previously calibrated with buffer solutions of pH 4.0 and pH 7.0. The ZP, PD, and PdI were determined using a Zetasizer Nano ZS apparatus (Malvern Instruments, Malvern, England.). For this purpose, samples were diluted in distilled water to a final concentration of 10% (*v*/*v*) and analyzed in triplicate. The datasets were submitted to ANOVA, and the means were separated by Tukey’s tests (*p* ≤ 0.05).

### 3.6. Morphological Evaluation of Nanosphere Suspensions

The homogeneity of the colloidal suspensions and the shape of the nanoparticles obtained were analyzed at the Microscopy Center of the Federal University of Minas Gerais [Universidade Federal de Minas Gerais-UFMG]. The colloidal suspension was deposited in 12-mm-diameter stubs, followed by silica drying. After total solvent evaporation, samples were metallized with 2 nm of Au/Pd metal alloy and subjected to scanning electron microscopy (SEM; Quanta 200 FEI, Thermo Fisher Scientific, Waltham, MA, USA). 

### 3.7. Stability Assay: Ultraviolet Light (UV)-Accelerated Degradation

The *Z. riedelianum* essential oil degradation assay was conducted in an ultraviolet chamber (BOITTON) coupled to a light containing two special lamps simulating radiation in the UV-A and UV-B spectrum with wavelengths of 365 and 254 nm, respectively. The system was kept at room temperature (25.0 ± 2.0 °C). A total of 1 mL of each essential oil sample (EO or NSEO) was transferred to clear vials and kept under UV radiation for 0, 0.5, 1, 2, 3, 5, 7, and 9 h. At each time interval, a 250-μL aliquot of each sample, in triplicate, was diluted in 1.5 mL (for NSEO) or 2.5 mL (for EO) of hexane for analysis. The mixtures were subsequently vortexed to obtain a liquid-liquid fraction, with the hexane and essential oil phase being analyzed by UV/VIS spectrophotometry using the method described at item 3.3.

### 3.8. Bioassays with B. tabaci Nymphs and Adults

The bioassays were conducted in the experimental area of Embrapa Rice and Bean in Santo Antônio de Goiás-GO, Brazil (16°30′24.57″ S, 49°17′06.53″ W). Whiteflies (*B. tabaci*) used in the experiments originated from Embrapa’s mass breeding program and were maintained on host plants such as common bean (*Phaseolus vulgaris*), soybean (*Glycine max*), and fava bean (*Phaseolus lunatus*). The *B. tabaci* species (Middle East Asia Minor 1-MEAM1, biotype B) was confirmed by mitochondrial gene analysis [11]. 

All bioassays were conducted in a screenhouse (18 m length × 7 m width × 4 m height) covered with anti-aphid (50 mesh) screen. Temperature, relative air humidity, and light incidence data were recorded using a data logger (AZ8829 data logger, AZ Instrument Corp. Ltd., Hong Kong, China) throughout the experiments.

The effect of the EO and NSEO *Z. riedelianum* fruit at concentrations of 0.25, 0.5, 1.0, and 1.5% were assessed on 2nd-instar nymphs, and on the repellency of adult whiteflies. As a positive control, the chemical insecticides cyantraniliprole (Benevia^®^, DuPont Crop Protection, Axis, AL, USA) and Oberon^®^ (spiromesifen, Bayer AG, Dormagen, Germany) were used in the assays conducted on nymphs and in the repellency tests, respectively. Both were used at a concentration of 0.25% (500 mL/ha) per the manufacturer’s recommendations. For the negative controls, distilled water, empty nanosphere suspensions (the same formulations used in the preparation of the nanoencapsulates, without essential oil), and Tween^®^ 80 emulsions at a concentration of 0.3% were used. The latter were used in the emulsification of EO.

### 3.9. Evaluation of Second-Instar Nymphal Mortality

Two experiments were conducted to evaluate the effect of EO and NSEO *Z. riedelianum* fruit on the mortality of 2nd-instar nymphs. Five seeds of the common bean Pérola cultivar (*P. vulgaris*) were sown in 1.5 L of soil (eutrophic red latosol) containing 2.8 g of NPK 5-30-15 fertilizer in polyethylene pots (1.5 L volume). After nine days, three seedlings, whose trefoils were sheared for primary leaf maintenance, were maintained per pot; the seedlings were subsequently infested with adult whiteflies. The whiteflies were kept on the bean stalks for 24 h for oviposition. After this period, the insects were removed using a handheld vacuum.

Nine days after the infestation date, during the second nymphal instar, each primary leaf was sprayed with 250 μL of each treatment on its abaxial face using a microsprayer (0.3 mm needle, Paasche^®^ H-series airbrush) coupled to a vacuum pump. 

A stereomicroscope at 40× magnification was used to evaluate live and dead nymphs in a delineated area (4 cm in diameter) in the central region of the abaxial face of the leaf. Evaluations began three days after spraying and were performed on every other day for two weeks. For each day of evaluation, one leaf from each plant was removed, for a total of four leaves per treatment. The evaluation criteria for dead nymphs included features such as dark coloration and dehydrated and wilted appearance. Empty puparia were used to determine the survival of the 4th-instar nymphs. Four replicates per treatment were used, with three plants per replicate containing two primary leaves each, in a completely randomized experimental design. The experiment was repeated over time. During treatment application, the conditions in the first experiment were (a) mean temperature = 23.4935 ± 2.94 °C, (b) mean relative humidity = 86.6595 ± 9.627%, and (c) mean light intensity = 480.85 ± 422.63 lm/ft^2^. The experimental conditions of the second experiment were (a) mean temperature = 24.5 ± 2.12 °C, (b) mean relative humidity = 48.41 ± 7.78%, and (c) mean light intensity = 259.5 ± 47.50 lm/ft^2^.

Nymphal mortality (%) was previously checked for normality and homoscedasticity with the Kolmogorov–Smirnov and Levene methods, respectively. All data were submitted to ANOVA and means were separated by a Scott–Knott test (*p* ≤ 0.05). 

For both experiments (1 and 2), dose-response curves for EO and NSEO were fitted using the 5-parameter generalized log-logistic model for a binomial response:

Experiment 1:

(4) $$y=c+\frac{d-c}{\left(1+exp\left(b\left(log\left(x\right)-log(e\right)\right))\right)f}$$

Experiment 2:

(5) $$y=c+\frac{d-c}{\left(1+exp\left(b\left(log\left(x\right)-e\right)\right)\right)f},$$

where *y* is proportional mortality, *b* the slope of the dose-response curve, *c* is the lower limit, *d* is the upper limit, *e* is the LC50 and *f* is the asymmetry parameter.

Dose-response curves were compared for parallelism among EO and NSEO using Wilcoxon’s non-parametric test at *p* < 0.05.

### 3.10. Repellent Effect of EO and NSEO Z. riedelianum Fruit on Adult Whiteflies

Two repellency tests were performed—(1) free-choice and (2) no-choice—using the same treatments described previously in the section “Bioassays with *B. tabaci* nymphs and adults”. In the free-choice test, all treated plants were placed together in the same cage and the females could choose the plant to oviposit. In the no-choice test, plants were kept individually in each cage. In both tests, seeds of the common bean cultivar Pérola (*P. vulgaris*) were sown in 400 mL of soil (eutrophic red latosol) in polyethylene pots (415 mL volume). After 7 days, only one seedling per pot was retained; each primary leaf was sprayed with 300 μL of each treatment on both leaf faces using a Paasche H-series airbrush connected to a vacuum pump. Four replicates per treatment were used, with three plants per replicate containing two primary leaves each, in a completely randomized experimental design. 

In the free-choice test, pots containing one seedling each were equidistantly placed in four cages (100 cm length × 45 cm width × 45 cm height) wrapped in voile. Each cage contained one replication of each treatment. A total of 350 adult whiteflies were released into each cage, where they remained for 24 h for oviposition. After this period, the leaves and insects were removed. Subsequently, the evaluation began with the number of eggs in a delineated area (4 cm in diameter) in the central region of the abaxial face of the leaf being counted with the aid of a stereoscopic microscope at 40× magnification.

In the no-choice test, each vase was placed in a cage (30 cm width × 50 cm height) wrapped with voile. A set of 30 adult whiteflies was released into each cage, so each group was exposed to only one type of treatment. After 24 h of oviposition, the insects and leaves were removed; the evaluation followed the same procedures described for the free-choice test.

The data set for number of eggs were previously checked for normality and homoscedasticity with Kolmogorov–Smirnov and Levene methods, respectively. All data were submitted to ANOVA. The mean number of eggs and oviposition index were separated by Kruskal–Wallis and “*t*” tests, respectively.

The deterrence test was evaluated by calculating the stimulant/deterrent oviposition index from the equation proposed by Fenemore [58], [(A − B)/(A + B)] × 100, where A = the number of eggs in the treatment to be tested, and B = the number of eggs in the control treatment. The index ranged from +100 (total stimulation) to zero (neutral) to −100 (total deterrence).

### 3.11. Statistical Analysis

Statistical software R version 3.1.2 (R Core Team 2014) was used for all analyses. 

## Figures and Tables

**Table 1:** Chemical composition of the *Z. riedelianum* fruit essential oil.

Peak	TR (min)	Constituents GC-MS	Exp. RI *	Lit. RI **	(%) GC-FID
1	5519	α-thujene	927	924	2.48
2	572	α-pinene	934	932	0.17
3	6784	sabinene	974	969	0.76
4	7241	β-myrcene	992	988	22.79
5	7708	α-phellandrene	1007	1002	0.53
6	8498	limonene	1029	1024	29.22
7	9137	β-ocimene	1047	1032	0.22
8	11,055	β-linalool	1101	1095	0.55
9	20,991	δ-elemene	1340	1335	0.66
10	21,496	α-cubebene	1352	1345	0.50
11	22,604	α-copaene	1379	1376	0.75
12	23,208	β-cubebene	1393	1388	0.72
13	23,285	β-elemene	1395	1389	0.45
14	24,416	β-caryophyllene	1423	1417	0.94
15	25,209	Aromadendrene	1442	1439	0.13
16	26,725	γ-muurolene	1480	1478	0.62
17	26,917	germacrene D	1484	1484	14.40
18	27,554	bicyclogermacrene	1500	1500	18.13
19	27,88	germacrene A	1509	1508	0.34
20	28,604	δ-cadinene	1527	1522	1.38
21	29,911	B germacrene	1561	1559	0.74
22	30,701	spathulenol	1582	1578	0.69
		Total			97.17

* Experimental retention index; ** Literature retention index.

**Table 2:** Precision (RSD%) ± SEM and accuracy (%) ± SEM of *Z. riedelianum* fruit essential oil samples used in the validation of the analytical method.

	Precision				Accuracy
Concentration (mg/mL)	Intraday 1	Intraday 2	Intraday 3	Interday	Interday
	(*n* = 3)	(*n* = 3)	(*n* = 3)	(*n* = 9)	(*n* = 9)
0.06	0.4± 0.002	0.4 ± 0.002	0.4 ± 0.002	0.4 ± 0.000	99.0 ± 0.3
0.15	0.3 ± 0.003	0.1 ± 0.001	0.4 ± 0.003	0.3 ± 0.153	100.3 ± 0.1
0.27	0.1 ± 0.002	0.0 ± 0.001	0.2 ± 0.003	0.1 ± 0.100	100.9 ± 0.5

**Table 3:** Particle diameter (PD), polydispersity index (PdI), zeta potential (ZP), pH, and encapsulation efficiency (EE%) of nanospheres (NS) containing *Z. riedelianum* fruit essential oil.

Formulations	PD * (nm)	PdI *	ZP * (mV)	pH *	EE%
NS 1	120.5 ± 8.77 *ab*	0.22 ± 0.01 *a*	−24.1 ± 5.8 *a*	6.66 ± 0.05 *a*	-
NS 2	106.7 ± 1.36 *b*	0.20 ± 0.02 *a*	−19.0 ± 2.4 *a*	6.48 ± 0.00 *ab*	98.82 ± 0.31
NS 3	117.8 ± 7.17 *ab*	0.22 ± 0.02 *a*	−24.5 ± 10.5 *a*	6.38 ± 0.04 *bc*	98.66 ± 0.05
NS 4	129.2 ± 6.96 *a*	0.23 ± 0.01 *a*	−26.6 ± 6.4 *a*	6.19 ± 0.11 *c*	99.31 ± 0.05

* Means followed by the same letters (*a*/*b*/*c*) in the same column are not significantly different according to Tukey’s test (*p* < 0.05). The composition of formulations is further described in Table 6.

**Table 4:** Mean number of eggs/leaf and oviposition index of *B. tabaci* after treatment of bean leaves with *Z. riedelianum* fruit essential oil (EO) and nanoencapsulated essential oil (NSEO) in free-choice and no-choice tests.

Treatments	Doses (%)	Eggs ^1^	Oviposition Index ^2^ (%)
**Free-Choice Test**			
EO	0.25	23 ± 17.59 *bc*	−40.26 **
	0.5	13.75 ± 8.97 *cd*	−59.41 **
	1.0	10.75 ± 6.12 *cd*	−66.80 **
	1.5	9.25 ± 5.85 *cd*	−70.75 **
NSEO	0.25	13.88 ± 10.67 *cd*	−56.73 **
	0.5	7.25 ± 5.38 *d*	−74.78 **
	1.0	9.62 ± 6.54 *cd*	−67.85 **
	1.5	15.12 ± 9.57 *cd*	−53.73 **
Water Control	-	40.38 ± 19.29 *ab*	-
Tween^®^80	0.3	54 ± 30.68 *a*	-
NS Control	-	50.25 ± 34.55 *ab*	-
Spiromesifen	0.25	11.12 ± 6.64 *cd*	−56.82 **
**No-choice Test**			
EO	0.25	5.62 ± 4.58 *e*	−51.09 **
	0.5	8.38 ± 4.66 *cde*	−34.95 **
	1.0	4.5 ± 5.43 *e*	−58.86 **
	1.5	4.25 ± 3.38 *e*	−60.69 **
NSEO	0.25	4.38 ± 2.44 *e*	−48.53 **
	0.5	7.75 ± 3.42 *cde*	−23.93 NS
	1.0	8.38 ± 6.5 *cde*	−20.24 NS
	1.5	7.5 ± 6.63 *de*	−25.47 NS
Water control	0	22.5 ± 12.99 *a*	-
Tween^®^80	0.3	17.38 ± 9.99 *ab*	-
NS control	0	12.62 ± 4.74 *abc*	-
Spiromesifen	0.25	11.38 ± 5.7 *bcd*	−32.82 NS

^1^ Means followed by different letters (*a*/*b*/*c*/*d*/*e*) are significantly different by the Kruskal–Wallis test (*p* < 0.05). ^2^ The oviposition index was calculated from the expression proposed by Fenemore [58], [(A − B)/(A + B)] × 100, where A = number of eggs in the test treatment, and B = number of eggs in the control treatment. For EO treatments, the Tween 80 treatment was used as comparison control. For NSEO treatments, the comparison control was empty nanospheres (NS control). For the insecticide control, the comparison control was water. ** Significant (*p* < 0.05).

**Table 5:** Percentage of dead *B. tabaci* nymphs after treatment of bean leaves with *Z. riedelianum* fruit essential oil (EO) and nanoencapsulated essential oil (NSEO) in two screenhouse experiments.

Treatments	Doses (%)	Experiment 1 (%) ^1^	Experiment 2 (%) ^1^
EO	0.25	56.53 ± 10.93 *b*	55.57 ± 18.87 *c*
	0.5	66.67 ± 11.07 *b*	64.30 ± 16.12 *c*
	1.0	81.80 ± 10.27 *a*	86.98 ± 8.04 *a*
	1.5	85.20 ± 1.12 *a*	91.23 ± 5.17 *a*
NSEO	0.25	30.23 ± 9.86 *c*	30.93 ± 5.81 *d*
	0.5	36.20 ± 7.02 *c*	31.20 ± 3.38 *d*
	1.0	66.13 ± 9.56 *b*	70.55 ± 13.78 *b*
	1.5	66.18 ± 10.8 *b*	82.87 ± 1.38 *a*
Water control	0	3.90 ± 2.94 *f*	1.45 ± 1.03 *f*
Tween^®^80	0.3	9.27 ± 4.69 *d*	10.82 ± 2.71 *e*
NS control	0	13.77 ± 0.85 *e*	5.16 ± 4.25 *e*
Cyantraniliprole	0.25	100.00 ± 0.00 *a*	100.00 ± 0.00 *a*

^1^ Means followed by different letters (*a*/*b*/*c*/*d*/*e*/*f*) are significantly different by the Scott–Knott test (*p* < 0.05).

**Table 6:** Composition of nanospheres containing *Z. riedelianum* fruit essential oils.

Formulations	PCL (mg)	Span 60 (mg)	Essential Oil (mg)	Acetone (mL)	Tween 80 (mg)	Distilled Water (mL)
NS1	150	50	0	10	50	20
NS2	150	50	50	10	50	20
NS3	150	50	100	10	50	20
NS4	150	50	250	10	50	20
